# Key discussions from the Working Party on Disorders of Sex Development (DSD) evaluation, Foundation Merieux, Annecy, France, March 14-17, 2012

**DOI:** 10.1186/1687-9856-2013-12

**Published:** 2013-07-08

**Authors:** Peter A Lee, Christopher P Houk

**Affiliations:** 1 Department of Pediatrics, Penn State College of Medicine, The Milton S. Hershey Medical Center, 500 University Drive, P.O. Box 850, Hershey, PA, 17033-0850, USA; 2 Department of Pediatrics, Georgia Health Sciences University, 1120 15th Street, Room BG1012, Augusta, GA, 30912, USA

## Abstract

Four topics from the DSD Working Party, a meeting to provide information regarding future studies, reported here are the complexities of hypospadias, surgical treatment of virilized genitalia of 46,XX DSD individuals, advances in phalloplasty and psychological, social and sexual outcomes.

## Background

The Working Party on Disorders of Sex Development (DSD) met in Annecy, France from March 15 to 17, 2012 to explore objective data sets to better describe the presentation, treatment and long-term outcome of patients with DSD. One goal was to identify objective data that could be validated and then applied internationally. Ideal outcomes would include normal adult phenotypes, functionality and quality of life. This conference aimed to identify the important issues concerning the evaluation of DSD patients, by assessing needs in three primary areas: diagnosis, treatment and outcome. This workshop was not a consensus meeting and was not designed to develop clinical guidelines, but rather sought to develop a status update on DSD, to be achieved by presenting and discussing relevant data. The Journal of Pediatric Urology [1] has published a supplement issue including referenced information from these three pertinent areas.

The Chicago consensus conference [2] was frequently referenced regarding diagnosis, medical/surgical treatment and outcome information in DSD. Over the past decade and particularly since the consensus conference, there continues to be a major shift in gender assignment of 46,XY DSD patients to a male gender assignment when there is evidence of testicular function and in utero androgen exposure [3,4]. Conversely, however, the failure of the consensus statement to address gender assignment for 46XX individuals with markedly masculinized external genitalia has largely led to the female assignment because of the interpretation of the default statement regarding sex of rearing for 46XX CAH patients with Prader 4 or 5 genitalia. Recent outcome information has provided a strong rational for consideration of a male assignment among such individuals [5,6]. Hence, this provides an example where the consensus conference notation of inadequate information is being supplemented by additional -- but as yet incomplete -- data.

The purpose of this publication is to discuss four main topics from this conference that may help to provide a broader perspective to practicing pediatric endocrinologists. These include; 1) complexities of hypospadias, 2) problems related primarily to the surgical treatment of virilized genitalia of 46,XX DSD individuals because of anatomic variability and difficulties of visualing and defining the internal anatomy, 3) advances in phalloplasty and 4) updated information on psychological, social and sexual outcomes of DSD individuals. Each section contains comments and suggestions relayed from the conference regarding next steps in improving this knowledge and treatment, particularly surgical treatment for children with DSD.

### Hypospadias

The Masculinization Programming Window (MPW) based on evidence from rodents is a period early in fetal life that determines phallic development and growth potential. Diminished masculinization may result in hypospadias and may be associated with a diminished anogenital distance (AGD), the distance between the anus and the edge of scrotum, in the male [7]. However, the pragmatic application of the impact of the MPW is unclear, including those with hypospadias since care is directed toward producing as functional and normal sized phallus as possible.

It is important to recognize that ‘distal hypospadias’ as defined by the urologist is not synonymous with what a pediatric endocrinologist would call ‘distal’ or ‘coronal; or ‘glanular’ hypospadias’. To the pediatric urologist, distal hypospadias indicates a phallus with a urethral meatus on the distal half of the penis. It is unclear which patients with distal hypospadias (as defined by urologists) will benefit from surgery not necessarily because of the programming of the MPW but because the status of underlying tissues cannot be determined until exploration at surgery. Hence, surgical approach and outcomes cannot be predicted. Current indications for surgical repair are presence of abnormal urinary stream and/or significant chordee. Once exploration has begun, if primary tubulization can be used, a low complication rate can be expected. While surgical techniques [8,9], are continually being refined, anatomic defects remain difficult to ascertain until visualization during surgery.

**Figure 1:**
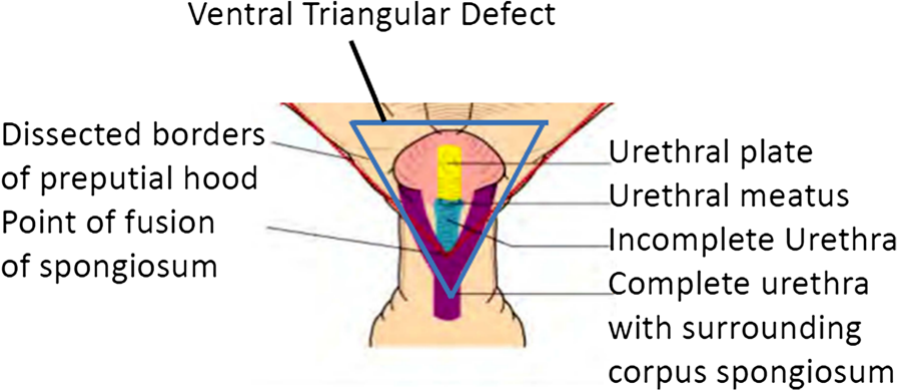
**Scheme of distal hypospadias with blue triangle outlining the defect of the urethral plate illustrating the more distal urethral meatus at a more distgal point than the point of fusion of the corpus spongiosum.** This later is not apparent on physical examination. Figure modified from several presented at meeting in Annecy.

This defect involving hypospadias of the distal half of the penis is associated with a narrow urethral plate that results from the lack of fusion of the corpus spongiosum overlying the urethra (Figure 1). Since the location of the urethral meatus is an inadequate surrogate of the severity of hypospadias, the underlying anatomy, and therefore the extent of repair required, cannot be inferred by visual or manual inspection. While the extent of this defect may be proportional to the chordee ranging from mild to severe, the extent of corpus spongiosum fusion can only be determined after degloving of the phallus at surgery. The point of fusion of the spongiosum, which often falls short of the point of urethral fusion at the meatus, can then be determined and the surgical approach planned. This area, the urethral plate, consists of a ventral triangular defect with its base at the existing urethral opening and its apex at the point where the corpus spongiosum prematurely divides in an area of fusion with surrounding dysplastic tissue; the urethral plate exists between the base and the apex of the triangular area (Figure 1). Beyond lack of fusion, development is considered good if the spongiosum is developed along the urethral plate with the urethra, but poor if there is a narrow, diminished spongiosum. The extent of fusion of the spongiosum and the overall quality of the urethral plate is used to determine the use of adjacent tissues for repair. Surrounding ventral tissues should be avoided if the urethral plate is dysplastic tissue of poor quality. If possible, dorsal tissue should be used. Post-operative growth of the hypospadiac penis suggests that postnatal growth of the penis is greater proximally than distally since the distal urethra grows at a slower pace after reconstruction of distal hypospadias.

Improved descriptions of this anatomy are not only necessary for optimal surgical repair, communication between healthcare providers may also allow for a more meaningful comparison between outcome studies. The extent of urethral fusion and underlying fusion categorized as proximal or distal division of the corpus spongiosum [10] together with a description of the surgery could provide a basis for both surgical guidelines and outcome assessment. Such information is needed for prospective long-term studies utilizing multivariate analyses [11] to permit comparison of the initial anatomy and surgical correction with anatomic, functional and psychosocial outcome considering the perspectives of surgeons, patients, family members and partners.

Interestingly, available outcome information does not support the concept that post-surgical normalization of genital anatomy leads to a satisfactory outcome. In fact, some with poor outcomes indicate satisfaction, implying that future studies should consider all aspects of normalcy from the patients’ perspective with comparison to other adult controls. Normal variation of the adult penis needs to be considered as the degree of penile curvature following hypospadias repair is similar to control men. In addition, studies need to account for the relatively large portion of the control population who do not have a urethral meatus at tip of the glans. A validated tool involves scoring (from 1 to 4) for 8 criteria: flaccid and erect penis size, penis thickness and meatal position, glans size and shape, penis appearance in general and testis/scrotum [12,13]. General dissatisfaction among repaired men who had hypospadias compared with normal men appears not to be related to anatomy as much as to sexuality. Poorer satisfaction among men who had hypospadias seems to be related to having fewer nocturnal emissions, fewer daytime sexual fantasies, less masturbation, and less foreplay and decreased frequency of sexual activity with fewer partners.

Urinary outcome reports also indicate general overall dissatisfaction relating to increased incidence of urinary tract infections, urinary stream problems and poor voiding control. Long-term outcome studies indicate more frequent complication rates than indicated by short term assessments and may be as high as 33% [14]. Complications including fistulas, urethral strictures and meatal stenosis become more prevalent the longer the duration of follow-up. Further, overall outcome including appearance, urinary and sexual function cannot be determined until after puberty.

The need for long-term, evidence- based outcome studies with uniform descriptions of defects at presentation and at surgery, the surgery itself and a multi-faceted assessment of adult anatomy, function, and quality of life is clear. A goal of this conference and the publication therefrom was to provide the basis for such studies^1^. It was suggested that standardized criteria be required for journal publication involving pre-operative anatomic description, any additional intraoperative revelations, detailed operative procedures, followed by a formalized outcome description. Outcome criteria would document cosmetic appearance compared with pre-surgical status, urinary flow and penile perception scores by the patient (and as age-appropriate-by the parents) based on questionnaire. Anatomic outcome details would include the straightness of erection, site of the meatus, quality of skin, any rotation of skin, glans configuration and peno-scrotal relationship and scrotal description.

The issues regarding hypospadias reviewed point out the key problem that the anatomic development of the complex development of the penile urethra cannot be determined before exposure at the time of sugery. Hence, the need for further research currently include a details description of the actual anatomy, involving primarily the development of the urethral triangle underlying the surface anatomy since the urethral meatus is often not indicative of the underlying development. Further, research needs to involve the description of new or previous surgical techniques with modifications, together with the various aspects of long term outcome regarding appearance and function.

### Virilized genitalia in 46,XX DSD individuals: anatomic description

#### Determination of anatomy of genital ambiguity

**Figure 2:**
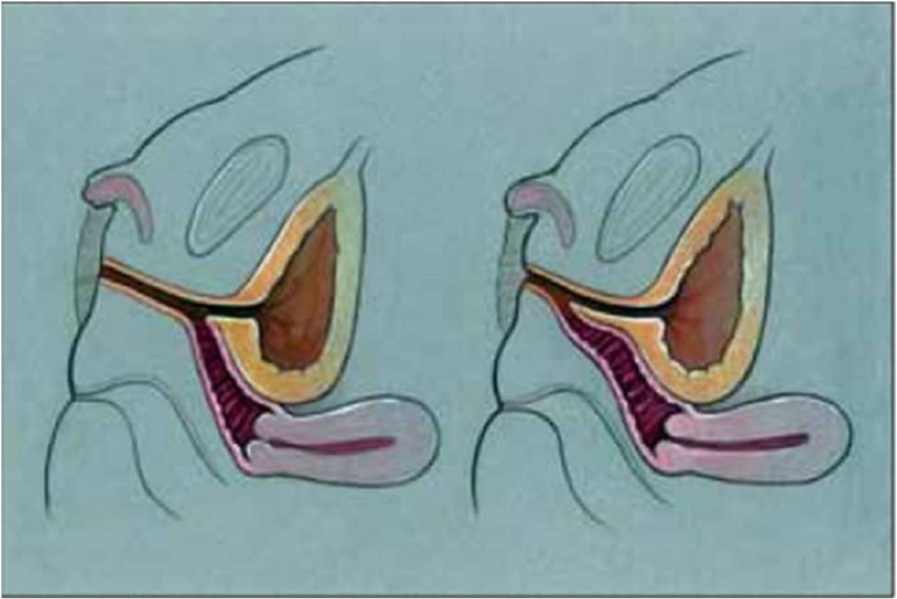
**Urogenital sinus anatomy: high (left) and low (right) confluence of vagina and urethra which exit as a common channel.** Unrestricted use from open access journal: Leslie JA, Cain MP, Rink RC. Feminizing genital reconstruction in congenital adrenal hyperplasia, Indian J Urol 2009; 25 (1):17–26.

While sonography of normal genitalia at 12 weeks gestation can differentiate male from female based on the angle of the phallus, even though phallic size differences between male and female are not present until late gestation. However, sufficient visualization of the internal reproductive anatomy in those with ambiguous genitalia is difficult and can be misleading at any age making preoperative planning difficult. The urinary outlet and vaginal introitus may each opening separately into the urogenital sinus or may create a urogenital confluence very high in the pelvis with the vagina opening into the urethra just below the bladder (Figure 2). Generally the more masculine the urethral formation, the more proximal and smaller the vagina but consistency between virilization as suggested by Prader staging and internal anatomy relationships cannot be assumed. Also, larger phallic size does not predict the extent of fusion of the labial folds. Hence, a detailed description strategy beyond Prader staging is needed.

#### Need for complete descriptions

In terms of both surgical decision making and generation of outcome study datasets it is critical that a complete description of the pre-surgical and post-surgical genitalia be documented including phallic size, the presence of 1 or 2 orifices, the configuration and extent of fusion, symmetry, and relative tissue quantity of both pairs of labial folds. Likewise, a complete description of internal anatomy includes visualization at onset of surgery documenting the presence and position of gonads, uterine size, vaginal length and location in relation to the bladder neck, urethra and urogenital sinus. Anogenital distance is an indication of extent of androgen exposure during early fetal life. The degree of masculinization of the bony pelvis should be documented since the vaginal angle may be altered enough to cause problems with intercourse. As described for hypospadias above, a record of all information preoperatively and during surgery provides the basis for the development of standardized protocols for treatment including surgery and key factors to be considered in outcome studies.

MRI or endoscopy may be useful in identification of confluence of the urethra and vagina, but genitography was not felt to be generally helpful. Ultrasound studies or MRI can be used for distance measurements for descriptions of the internal phenotype. Length and width of the urogenital sinus and visualized internal structures such as the uterus should be included. However, the location of the urethral sphincter, an important structure to be avoided at surgery, usually cannot be determined.

Use of standardized data sheets could provide uniform recording of information including prenatal assessment, initial post-natal and subsequent pre-operative evaluation, findings and techniques at surgery and at specified time points for years or even decades after surgery would provide the basis for recommended clinical approach and outcome studies. Outcome data should include whether excess clitoral tissue used for concomitant vaginoplasty, the age of vaginoplasty, any repeat surgeries required, and the use of vaginal dilation without surgery or after vaginal reconstruction; vaginal dilation was considered inappropriate until after puberty. The role of estrogen upon healing after surgery; short-term estrogen therapy for the prepubertal child or in the estrogenised pubertal female has been suggested [15], the use of administered estrogen or the clinical evidence of estrogenization at surgery should be recorded.

An aim of prospective outcome studies is to clarify how complications are related to pre-surgical anatomy and surgical techniques. Surgical techniques vary from flap vaginoplasties which have been recommended for those with low junction of the vagina and urethra in which the urogenital sinus may be left intact; to vaginal pull-through procedures which are difficult when using a perineal approach, and may involve partial mobilization [16] or total mobilization [17,18], which commonly results in a low bladder neck; and an anterior surgical-transrectal approach [19] for the high urogenital sinus. After clitoroplasty, information concerning the preservation of Buchs fascia with the neurovascular bundle and histologic description of nerves within removed erectile tissue are pertinent. Since the goal is normal appearing genitalia with a vagina adequate to admit 2 fingers and greater than 6 cm. depth at the end of therapy, this information should be included in outcome datasets. Surgical outcome evaluation for vaginoplasty includes satisfaction with sexual function, and comparison of the utility of skin grafts versus intestinal mucosal replacement. The former has been suggested to be problematic because of lubrication difficulties and hair growth when hair-bearing skin is used while excessive mucus secretion is a complaint when intestinal mucosal lining is employed. At present, outcome studies suggest that urinary complaints are more common following vaginoplasty than occurs in the general population [20], it is possible that future outcome studies will help to refine surgical techniques and postoperative treatments to improve vaginoplasty outcomes.

#### Decisions regarding age of surgery

The decision regarding surgery and the age of surgery are informed by internal anatomic relationships. If both orifices open into the urogenital sinus, surgery may be deferred until a much older age, depending upon other factors. For the patient with CAH with high confluence, it is generally, although not universally, accepted that early surgery in infancy should be considered because the distance to the perineum is less, there is likely to be adjacent available tissues to use and it is generally technically easier. There was no agreement regarding the criteria for early or late genital surgery, particularly vaginoplasty. Suggested considerations include a potential for better wound healing after surgery during infancy, the use of urogenital tissue with early clitoroplasty [21] particularly if the urogenital sinus is long, the perception that early surgery is technically easier with better outcome for those with high vaginal insertion into the urethra and higher urogenital sinuses [22]. The likelihood of the need for repeat surgery is greater after early surgery even though such surgery may be relatively minor. If surgery is not done as an infant, it is generally considered that it should not be done until adolescence. Currently, a delay of surgery is recommended until adolescence for an absent or foreshortened vagina as present in the patient with Rokitansky or Complete Androgen Insensitivity Syndromes. For the cloacal extrophy group, vaginal surgery should be done at the time of ano/cloacal rectal surgery.

#### Goal of improved outcomes

It has been accepted that in the past some genital surgeries done in childhood have resulted in poor cosmesis and reduced genital sensitivity leading to adult sexual dysfunction. While current nerve sparring feminizing surgery is expected to enjoy better outcomes regarding appearance and genital sensitivity [23], long-term outcomes supporting this have not yet been documented. Based on follow-up data in DSD patients who underwent genital surgery in childhood, common complications include scarring and stenosis; in those with CAH who did not have ideal adrenal suppression, scrotalization is common and, if skin was used for vaginoplasty, hair in the vagina. If surgery resulted in reduced sensation, this negatively impacts sexual function [24]. Currently, surgical techniques involve corporal sparing clitoroplasty with, if necessary, glans reduction using techniques such as a subtotal de-epithelization-wedge. The approach considers external genital proportions [25], before surgery with appropriate proportions created or maintained after surgery.

It is anticipated that outcome of those DSD individuals having surgery currently will have better outcomes when compared to previous surgical techniques because of improved surgical techniques and a better appreciation of the female sexual response. Such outcome needs to be documented using a multifactorial approach as discussed earlier.

Future research needs involve careful documentation of decision to proceed with or delay surgery, together with descriptions of surgery, together with details outcome information. As with hypospadias above, much of this information must involve the surgeon and hence this will be the primary group involved in these studies.

### Phalloplasty

Historically, a major limitation in sex assignment of 46,XY DSD patients with phallic deficiency was the problem of surgically creating a functional penis. This limitation often resulted in a female assignment since surgery to create a vagina was considered likely to be more successful. Current surgical advances when care is taken to preserve innervation and sensitive tissues have resulted in the ability to create a more functional phallus.

**Figure 3:**
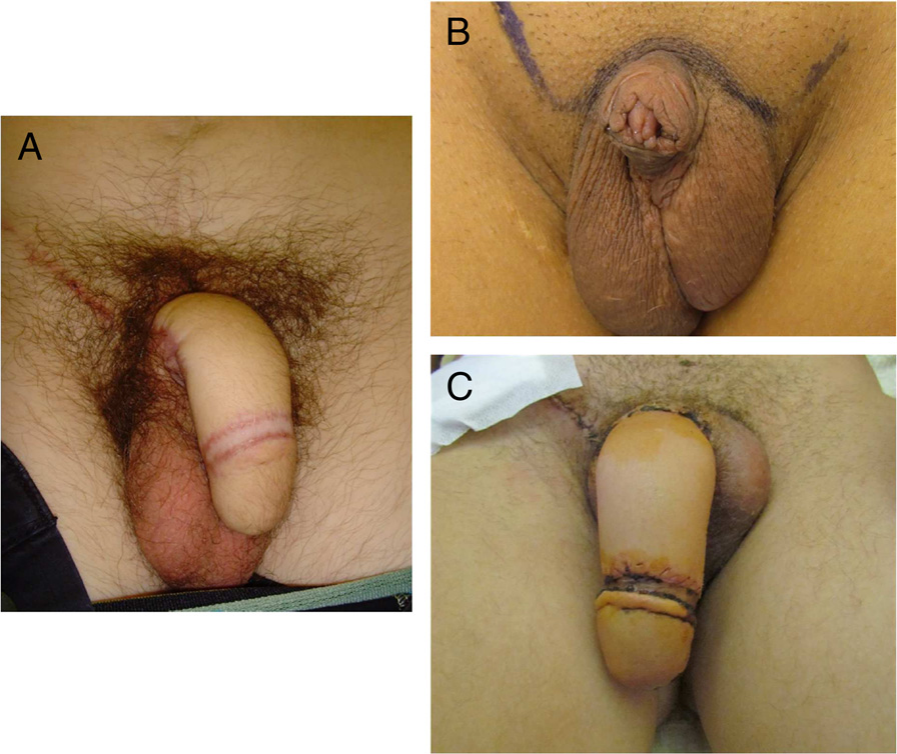
**Results of phalloplasty for 2 46,XY boys.** Genitalia of: **A.** Exstrophy patient who lost his penis during reconstructive surgery, **B.** Preoperatively of Partial Androgen Insensitivity Syndrome (PAIS) patient with an insufficient penis after multiple hypospadias repairs. **C.** Post-operative PAIS after phalloplasty and subsequent implant one year later. Kindly provided by Piet Hoebeke, Department of Urology, University Hospital, Gent, Belgium.

Most of these significant surgical advances have been made due to the demand for phalloplasty [26,27], from female to male transsexuals. Prostheses have erectile capabilities and attempts to connect nerves for erogenous sensitivity and proprioception. Donor sites include arm and thigh tissues, with an option of the use of separate donor sites for the urethra from the hairless, ventral side of the forearm. Candidates for surgery include those with penile agenesis, dysfunctional or “cripple” exstrophy, “cripple” hypospadias and epispadias, circumcision accidents, micropenis, small penis with DSD diagnoses and “shriveled” penis (<6 cm erect length). Figure 3A shows the genitalia of an exstrophy patient who lost his penis during reconstructive surgery and who was offered a phalloplasty at age 16 years with implantation of erectile device 1 year later. Currently, he is a 20 year old student who his extremely happy with his phallus, has a girlfriend and lives like any other adolescent of his age. Figures 3B and 3C show pre- and post-operative genitalia of three brothers with Partial Androgen Insensitivity Syndrome (PAIS) who underwent phalloplasty and subsequent erectile implant. Before surgery, multiple hypospadias repairs resulted in a insufficient penis.

Evidence suggests that outcomes are improved when existing penile structures or other sensitive genital tissues can be incorporated into the prosthesis. Penises smaller than 6 cm can be incorporated into the prosthesis while that > 6 cm cannot. Outcome is less favorable in extrophy when the poorly developed penile structure cannot be incorporated. The basic approach involves keeping and using any tissue that is erectile plus all tissue that is sensitive. Preoperative assessment includes determination of whether tissues, such as any corpora, can be lengthened. Surgery after puberty is best since sexual sensitivity can be assessed and the patient can be fully involved in all decision making.

Outcome research for those having phalloplasty, as for the other post-surgical issues described here must involve the initial genital anatomy, description of which tissue were used for construction of the phallus as well as the reconstruction procedure, together with long term functional and cosmetic satisfaction.

### Psychological, social and sexual outcomes

Previous evaluations of DSD psychological and gender issues have often involved case histories rather than case–control studies. These have shown that important information concerning their condition may be unknown to the patients, such as a married 46XY female who believed that her previous feminizing surgery had caused her infertility. It is important to note that this may occur even when attempts toward full disclosure and psychological support are made. Children develop their own ideas of what is normal. Future studies need to assess not only an individual’s basic knowledge of his/her condition but also the patient’s perception of if and how they are different and how they perceive why they are different.

Particularly among those who had sex assignment in infancy, it is important to consider apparent gender identity among the young as provisional while preparing parents for potential, but unlikely, gender change. Gender change, a consequence of both pre- and post-natal biological factors and social factors, must always be patient-initiated. When gender issues are unclear, as for example in a pubertal-aged DSD person being raised female and a potential need for gonadectomy; one should precede cautiously before considering irreversible surgery. A temporary option is GnRHa suppression, as used for similarly aged transsexual persons. Patients at this age or older need to ponder their perception of gender assignment since it appears that fewer 46,XY DSD individual have gender questions over time, with the incidence decreasing to about 12%. It has long been noted that among 46,XX CAH females, while there may be significant overlap in feminine-masculine gender role behaviors that gender identity is usually female indicating that many of the social cues commonly used to indicate gender, such as gender role behaviors, are not closely aligned with gender identity outcomes.

There is more psychological, social and sexual outcome information among 46XX CAH females that other condition. Differences in play behavior and toy preference during childhood are well documented [28]; as is involvement in male dominated professions as adults [29,30]. These differences are apparently influenced by both intrinsic and environmental factors. Outcome information also indicates that adult 46XX CAH females often live alone, have fewer social contacts and are less likely to be employed than age-matched peers [31]. An increased incidence of sexual dysfunction is also present and appears to be related to genital insensitivity, difficulty/inability to orgasm, atypical genital appearance and decreased fertility [32]. Table 1 summarizes some findings suggesting that dysfunction is related to both the severity of the underlying condition (based on genotype) as well as surgery. It is of interest that the ability to achieve orgasm does not differ from control women and does not appear to correlate with sensitivity and that as a group sexual life satisfaction does not differ between CAH women and controls nor does it correlate with ability to orgasm. In addition, this report also provides preliminary evidence of better outcome with the currently used nerve sparing clitoroplasty when compared to older techniques.

**Table 1:** Sexual Function among CAH Women: Relationships with genotype* and surgery

**Sexual function**	**Based on genotype and surgery**
Clitoral sensitivity	Highest for nerve sparing and clitoral placement
	No difference between no surgery and partial resection
	Poorest with subcutaneous clitoral placement/partial resection
Ability to achieve orgasm	Did not correlate with sensitivity
	Not different between genotype groups or from controls
Sexual function score	Score correlated with severity of genotype
(9 aspects of sexual life)	Lowest in null phenotype group
	Lower in null and 12-splice phenotype than controls
Satisfaction with sexual life	No difference between CAH women and controls
	CAH group with null phenotype (who had more surgical complications) differed from other phenotypes
	Satisfaction did not correlate with ability to orgasm

*CYP21A2 allele mutations: null, 12-splice, I172N and V281L.

The psycho-sexual impact of genital surgery involves both an individual’s perception of his/her genitalia before surgery and the impact of surgery on genital sensitivity and the presence of actual or perceived change in genital appearance. Most of these are factors that are impacted by current and previous positive or negative social and environmental influences. Sexual function can be hindered by actual or perceived genital differences and anatomic and sensual physical limitations. Stigmas including fear, guilt, and shame may lead to avoidance of situations that could lead to sexual activity. A recent review of ninety eight 46,XX DSD CAH patients suggests that psychological problems result in negative outcome in 68% most of whom blame prenatal androgen exposure [33]. This group was also found to have less sexual experience, more sexual anxiety, more behavior avoidance, and more passivity compared with type 1 diabetes mellitus patients [34]. Also among XY women who had vaginoplasty, a poor understanding of their development and lack of a sense of entitlement regarding sex was found to be associated with limited social interaction including even a lack of engagement conversations simply for pleasure.

With the latest medical and surgical care, disclosure and counseling are crucial for good outcomes. Such must focus upon the underlying conditions and expected gonad function and will differ for those with hormonal (cAIS, CAH, 5alpha, etc.) versus non-hormonal DSD (bladder/ cloacal exstrophy, epispadias, aphallia, and vaginal agenesis). Learning disabilities and a higher rate of internalizing psychopathologic disorders with risks for suicide and behavior aberrations have been identified in DSD patients and if suspected, appropriate evaluation including neuropsychologic testing should be considered. Before proceeding to full disclosure, careful consideration is needed and agreement from the individual is pertinent, since full medical disclosure is not always positive at all times. Regarding sexual issues, a desire for sexual satisfaction should always be assumed. If surgery is planned, its impact on current or future mates should be discussed. Learning how to be verbally intimate is important before approaching physical intimacies.

Psychosocial wellness involves being self-determined, resourceful, and knowledgeable, feeling good enough for intimacy and finding actual experiences pleasurable, leading to enjoyment. Predictors of good outcome involve lack of stigma related to body image and genital perceptions, knowledge and understanding of condition, attitudes and aspirations, general physical and mental health, understanding and outcome of surgical interventions, coupled with social interactions and impact of education, culture and religion. Assessment with Quality of Life indicators based on judgment of patients satisfaction and achievement and sensitivity to life situations including sexual experiences are crucial. Such may reveal individual’s unawareness or suppression of negative factors (WHOQOL) [35,36]. Future research needs to involve prospective observational studies with repeated measure design within bio-psycho-social models, be hypothesis-driven and will likely require non-standardized questionnaires.

Long-term psychosocial and psychosexual outcomes are highly complex and while details studies must involve assessment of qualify to life outcome information, multifaceted analyses are needed to correlate outcome with initial development issues, psychological and social support and surgical aspects, if involved.

## Conclusion

A primary goal of this conference was to provide a format by which multi-centered outcome studies could be conducted. This report focuses upon four areas: distal hypospadias, genital assessment and repair for virilized 46, XX CAH patients, potential for phalloplasty and psychological, social and sexual outcomes study perspectives. Each continues to be problematic regarding assessment and therapy and careful outcome studies are needed for each, to assess past therapy and improve it in the future.

## Consent

Written informed consent was obtained from the patient for the publication of this report and any accompanying images.

## Competing interests

The authors declare that they have no competing interests.

## Authors’ contributions

Both authors took extensive notes of the sessions of the working conference on disorders of sex development and both incorporated these notes into this meeting report. Hence, both have written, read and approve the final manuscript.
